# Long-Term Effects of a Kinematic Bikefitting Method on Pain, Comfort, and Fatigue: A Prospective Cohort Study

**DOI:** 10.3390/ijerph191912949

**Published:** 2022-10-10

**Authors:** Robson Dias Scoz, Paulo Rui de Oliveira, Cleyton Salvego Santos, Júlia Ribeiro Pinto, Cesar Augusto Melo-Silva, André Filipe Teixeira de Júdice, José João Baltazar Mendes, Luciano Maia Alves Ferreira, César Ferreira Amorim

**Affiliations:** 1 Programs in Physical Therapy, University City of Sao Paulo (UNICID), Sao Paulo 05508-030, Brazil; 2 Medical Sciences Program, Brasilia University, Brasilia 70910-900, Brazil; 3 Physiotherapy Department, University Hospital of Brasília, Brasilia 70840-901, Brazil; 4 Interdisciplinary Investigation Center Egas Moniz (CiiEM), Laboratory of Physical and Functional Assessment in Physiotherapy (LAFFFi), 2829-511 Monte de Caparica, Setubal, Portugal; 5 Research Laboratory BioNR, Physical Therapy Department, Quebec University, Saguenay, QC G7H2B1, Canada; 6 Lab Corinthians R9, Sport Club Corinthians Paulista, Sao Paulo 03828-000, Brazil

**Keywords:** kinematics, bicycling, mountain biking, sports equipment, bike-fit, comfort, pain, fatigue

## Abstract

**Highlights:**

**What are the main findings?**

- Pain and comfort levels improved after bikefit and remained stable through 4 months.
- Even with discrete increase after 3 months, fatigue levels remained significantly lower.

**What is the implication of the main findings?**

- Ergonomic adjustments through bikefitting improves riding experience for a long period of time, and it can contribute to increase cycling adhesion.
- With increased riding comfort and reduced pain, participants showed increased fatigue and mileage levels, indicating increased sports practice.

**Abstract:**

The purpose of this study is to analyze the long-term riders’ subjective responses to a standardized bikefitting method on their bicycles. Eighty-six amateur mountain bikers had their riding posture and bicycle components ergonomically adjusted through a 3D kinematic bikefitting method. Validated subjective scales (Feeling, OMNI, and Numerical Rating Pain Scale) were used to assess their overall riding comfort and fatigue along with localized pain for six body parts. Data were collected just before intervention (baseline or pre), immediately after (or post), and 30, 60, 90, and 120 days after the bikefit session. A Student’s *t*-test comparing before bikefit and after 120 days showed significant (*p* < 0.05) reduction in localized pain for all six body parts and riding comfort along with a large effect size effect (*d* = 1.18) for riding comfort. Although initially reduced, fatigue scores gradually increased over the months, showing a high correlation (r = 0.946) with increased monthly training volume. In conclusion, overall riding discomfort and pain were significantly decreased after a standardized kinematic bikefit session even after 120 days post intervention. However, fatigue scores began to rise after 30 days, showing a high correlation with increasing monthly training volume.

## 1. Introduction

Cycling has been chosen as a mode of transportation, recreation, and sports modality for thousands if not millions of people around the world; however, this increase in popularity has been increasing the incidence of musculoskeletal non-traumatic injuries [1]. These are usually due to incorrect riding posture or technique but also due to inadequate bicycle components setup according to its rider’s anthropometric characteristics, flexibility, and previous musculoskeletal injuries [2,3,4,5,6,7]. Riding pain or even discomfort can lead a professional cyclist to lose athletic performance, while beginners trying to adhere to a active life style through cycling may abandon the sports practice [4,6,8]. Strategies aiming to enlarge and improve cycling practice could positively impact not only individual health (reducing sedentarism) but also contribute to other social and economic areas such as urban traffic and environmental pollution [9].

The ergonomic process comprising bicycle adjustments (also known as “bikefit” or “bikefitting”, thus calling its professionals “bikefitters”) emphasizes riding comfort improvements by adapting bicycle components according to its rider’s complains and objectives. The focus of the process is to reduce or eliminate pain generated from repetitive musculoskeletal overloads. Painful body parts are usually produced after long, light-to-moderate cycling exercise sessions or after short, high-intensity cycling exercise sessions but both with an inadequate bicycle configuration in relation to the rider’s anthropometric characteristics, flexibility, and previous musculoskeletal injury history [3,10,11].

As some studies have pointed out a possible performance improvement after bicycle ergonomic adjustment [6,10,12,13,14,15,16], these studies have supported the work of bikefitters along with professional athletes to improve their athletic performance on formal competitions. On the other hand, few studies have given attention to a cyclist’s adherence to sports practice when faced with riding discomfort or pain. Both are considered fundamental aspects to amateur cyclists, such as beginners and commuters [17,18].

The majority of scientific studies concerning riding posture focus their attention on knee kinematic and kinetic analysis, as some studies have shown a high injury prevalence and incidence on that joint [19,20,21,22]. However, several other musculoskeletal non-traumatic injuries (such as back pain and genitourinary problems) are reported by recreational and professional cyclists alike [23,24]. A more ergonomically adjusted bicycle could reduce the severity of those injuries, improving overall riding comfort while reducing repetitive overloads. To that end, a rider whole-body kinematic approach is needed, as these data can guide the selection of better joint angular ranges during pedaling [1,21]. Through whole-body kinematic data (i.e., riding posture), cyclists could decide the best configuration of their equipment to avoid overload musculoskeletal injuries and even to improve their cycling performance [4,25,26,27].

One recent study on bicycling kinematics indicated an improvement in comfort and pain reduction on cyclists that were subjected to a kinematic bikefitting method using the rider’s joint angular measurements as guidelines [28]. However, our results showed only short-term effects, so it is unknown if these improvements would endure through the passage of months after the bikefitting session. After a long scientific database search, we could not find a study to answer this question. Even the concept of what could be considered long-term effects of bikefitting procedures could not be found. Thus, the subject is still under scientific interest, as bikefitters, physiotherapists, coaches, and athletes could benefit from those answered questions.

This study aims to examine the subjective reactions of mountain bike riders to an ergonomic alteration made to their bicycles. Our hypothesis is that even after 120 days from intervention, the levels of subjective pain, discomfort, and fatigue will remain lower than baseline.

## 2. Methods

### 2.1. Design

This is a prospective cohort study partially based on data from a clinical trial currently under the reviewing process. This research followed recommendations of several ethics and methodological guidelines [29,30,31,32]. The study procedures were approved by our university Research Ethics Committee with the protocol #4442645. An informed consent form, containing this research information along with risks, benefits, and purposes, was voluntarily and individually signed by all candidates before being included in the study sample. Each author certifies that they had no competing interests during conducting this study. Patients or the general public were not involved in the planning, execution, reporting, or distribution of this study.

### 2.2. Participants

The sample size calculation was based on our previously published experimental study concerning cycling kinematic analysis and subjective scales [28]. In that study, we used an alpha level of 5%, loss of follow-up limited to 10%, and power of 90% to find that 76 participants would be necessary. All calculations used G* Power Software version 3.1.9.6 (University of Dusseldorf, Dusseldorf, Germany) [33].

Advertisements were posted online on social media and in bicycle stores to recruit candidates. They were instructed to fill out an online form with contact information for a future interview with a member of the study team. Bicyclists (or mountain bikers, MTB) had to be regularly enrolled in cycling activities with their own mountain bike for the previous three months, at least three times per week, to be included in this study.

Candidates with osteo-muscular injuries that would prevent them from participating in sports were not included in the sample. The use of any analgesic-inducing medicine or receiving current pain treatment were additional criteria for exclusion from the sample; cyclists younger than 18 or older than 60 years old; with less than a month of experience with its current bicycle; and those with the absence of any discomfort or musculoskeletal complain while riding their bicycle were also excluded. Cyclists who had recently undergone a surgery were in current physical rehabilitation, or who responded “yes” to two or more items on the Physical Activity Readiness Questionnaire (PARQ) [34] were eliminated from selection, as it could indicate cardiopulmonary risk during exercise.

The candidates received an informed consent form to agree with and confirm participation in the study during the interview, which included an explanation of the study’s objectives, experimental methods, potential risks, and advantages. To reduce the danger of data loss or leakage, the final data were kept on a password-secure, cloud-based website. The final sample was formed by 80 amateur, adult mountain bike cyclists (54 males and 26 females), classified as recreational (*n* = 19) and competitive (*n* = 61) according to a recent cycling categorization based on weekly training/practice volume in kilometers [19]. All participants declared being amateur cyclists and as not receiving any financial support for practice of the sport.

**Figure 1:**
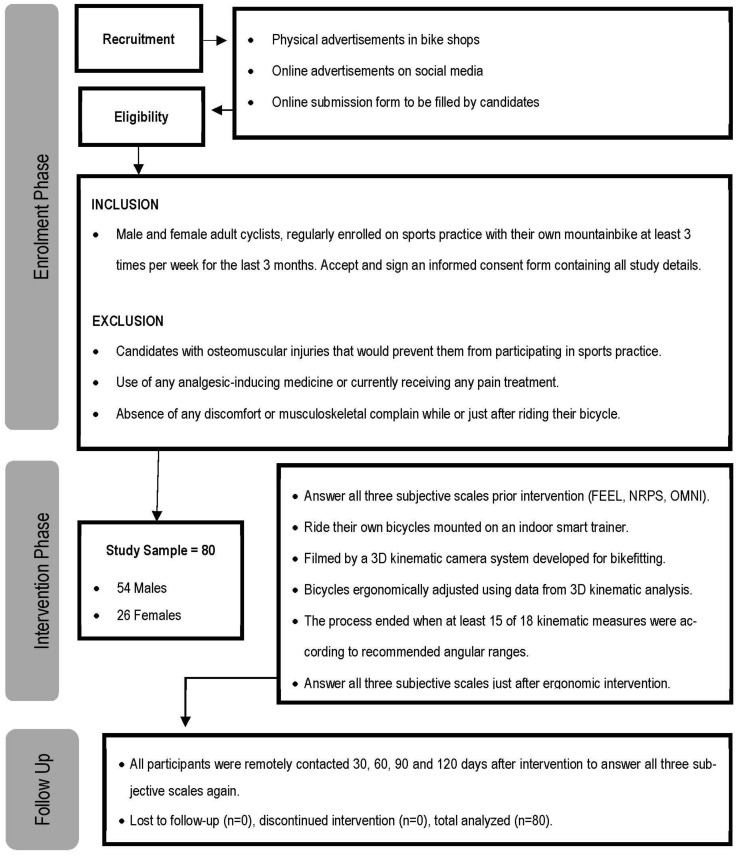
Study workflow.

Demographic and anthropometric information of the sample is presented in Table 1, while Figure 1 shows a schematic workflow of participants enrollment and study processes.

### 2.3. Instruments

For data collection, scientific validated [35], LED-emitting infrared tri-dimensional camera system (Vantage Camera System, Retul Inc., Boulder, CO, USA), commonly used in commercial bikefitting (also known as Retul 3D Cameras), was used. Calibration of the system followed the manufacturer’s manual instructions. The professional responsible for all bikefitting sessions has 10 years’ experience and an advance training with the camera system and its body-marker placement protocol.

Each participant’s bicycle was attached to a hydraulic indoor direct-drive smart trainer with a built-in power meter (Suito Smart Trainer, Elite, Fontaniva, Italy). Common mechanical tools (such as screwdrivers and hex keys) were used to change and adjust bicycle parts (using kinematic data as a guideline), aiming to improving rider’s posture.

Three validated subjective scales were used to acquire the primary outcome measures: an overall riding comfort scale (Feeling Scale, or FEEL) [36], an overall riding fatigue scale (OMNI Scale, or OMNI) [37], and a numeric rating pain scale (NRPS) [38]. The NRPS was used for six body parts as common sites of physical complains while pedaling (hands/wrists or HW, neck/shoulders or NS, back/hips or BH, groin/pelvis or GP, knee/thigh or KT, ankle/feet or AF) [1].

We used a MacBook Pro Notebook (Cupertino, CA, USA) for data storage and processing. It was outfitted with the Microsoft Office for Mac software version 2011, Jamovi Software (Sydney, Australia), and IBM’s Statistical Package for Social Sciences (SPSS) (Armonk, NY, USA).

### 2.4. Intervention Procedures

**Figure 2:**
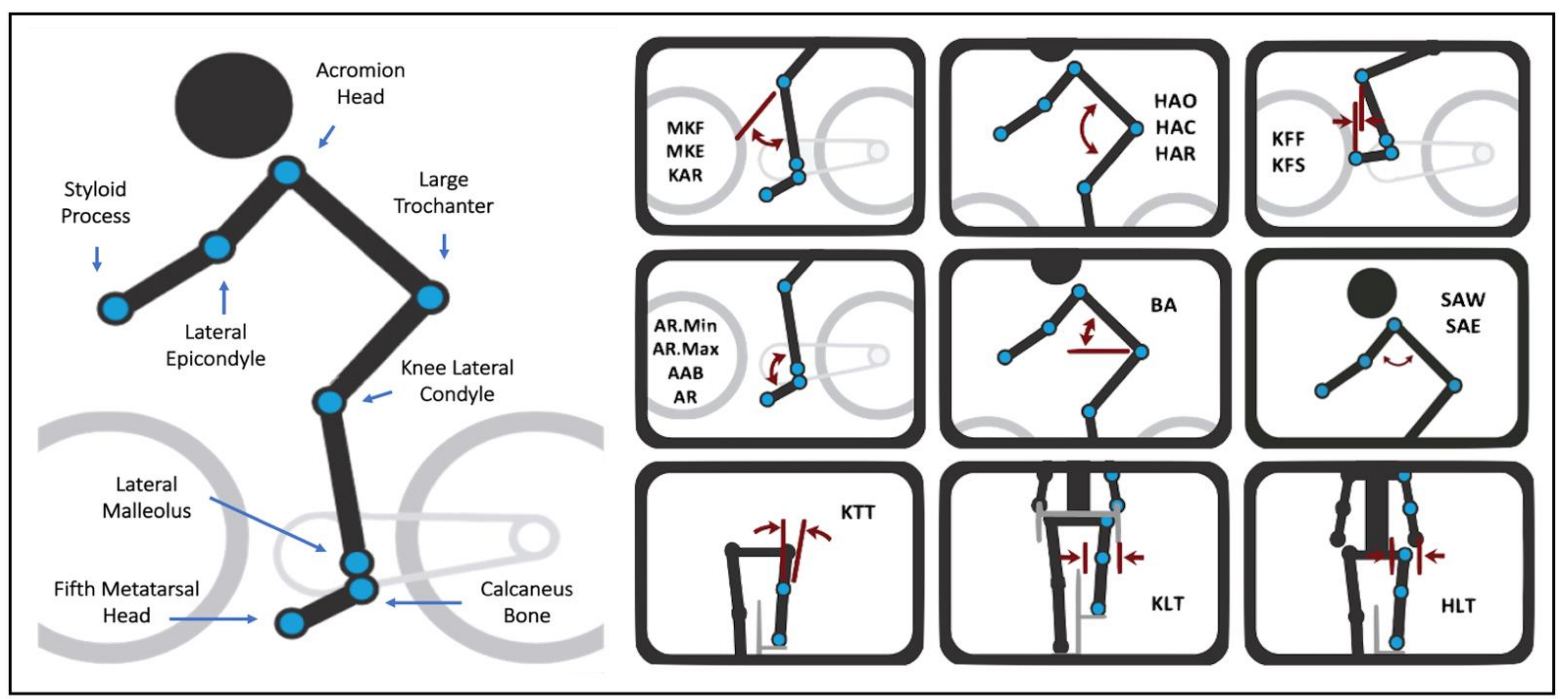
Schematic layout of all 3D kinematic measurements used with rider’s body markers location. Ankle minimum (AR.Min), ankle maximum (AR.Max), ankle range (AR), ankle angle at bottom (MB), maximum knee flexion (MKF), maximum knee extension (MKE), knee angle range (KAR), knee forward of foot (KFF), knee forward of spindle (KFS), knee travel tilt (KTT), knee lateral travel (KLT), hip angle closed (HAC), hip angle open (HAO), hip angle range (HAR), hip lateral travel (HLT), back angle (BA), shoulder angle to wrist (SAW), shoulder angle to elbow (SAE).

A standardized bikefitting procedure based on 3D kinematic data was applied to the subjects. Bicycle component modifications were guided by reference values for joint angles, riding posture, and spatial relationships with bicycle geometry. Ranges of reference joint angles were gathered from our previously published experimental study with 160 mountain bikers, where those joint angles generated (although at short-term) significant riding comfort and pain reduction while pedaling [28]. Figure 2 shows all 18 reference values used during the 3D kinematic session with the respective measurements’ descriptions. All measures are schematically laid out using all rider body markers.

At a convenient pre-scheduled time, participants were instructed to bring their bicycle to the lab. They were given a list of suggestions, which included dressing appropriately for cycling and wearing appropriate footwear; refraining from vigorous exercise shortly before the bikefit session; and avoiding hunger, sleep deprivation, and dehydration. They could request fresh water at any time during the meeting. The inside temperature was kept at 23 degrees Celsius, while the relative humidity ranged from 60 to 80 percent. Both the riders’ right and left sides were captured by Retul 3D camera system.

Participants received a thorough explanation and demonstration of all laboratory processes when they arrived. The cyclists’ personal information, amount of bicycle expertise, weekly riding mileage in kilometers, and personal goals, expectations, and complains were all registered (when they had to answer all three subjective scales—NRPS, FEEL, and OMNI). PRE was the name given to this baseline data collection. The International Society for the Advancement of Kineanthropometry (ISAK) Level 01 accredited anthropometrics protocol [39] was used to record anthropometric data.

After interview and physical examination, each subject started to ride their bicycle on the smart trainer for 120 s at 70–90 rpm with an automatically controlled load of 100 watts. As amateur mountain bikers, 77% of our sample considered this training load and cadence similar to their experience of outdoor cycling. In addition, this riding cadence was considered usual for that population, according to other authors [40]. After 120 s, participants could dismount from the bike and relax while ergonomic changes to their bicycle were made using suggested joint angular ranges (Table 2). The session was over when at least 15 out of the 18 measurements fell within the recommended ranges. At that moment, the subject answered the three subjective scales again, and we called these data extraction POST.

When the subjects were released, they were told not to alter any bicycle parts or measurements for 120 days. We contacted each participant on follow-up time-points called 30D (30 days after bikefitting), 60D (60 days after bikefitting), 90D (90 days after bikefitting), and 120D (120 days after bikefitting). Each cyclist was instructed maintain their weekly mileage during this period to report the most accurate impression of the bikefitting process’s long-term effects. We contacted each participant remotely to answer all three subjective scales at each time point.

### 2.5. Data Analysis

Sex, age, height, weight, wingspan, BMI (body mass index), experience (familiarity) with the current bicycle in months, and rider training (practice) volume in kilometers per month were the demographic and anthropometric data retrieved. Table 1 displays our sample demographic and anthropometric information.

Feeling Scale (FEEL) values, Visual Analog Pain Scale (NRPS) values, and OMINI Scale (OMINI) values were collected at six moments: during each participant session appointment (pre, or baseline), immediately after the intervention (post), and 30, 60, 90, and 120 days after intervention (follow up). These data were gathered for inferential analysis. We applied an intention-to-treat analysis to all data.

The Kolmogorov–Smirnov test and subjective inspection were both used to validate the normality of all data. Using Levene’s test, the homogeneity of variance was evaluated. To find statistically significant variations between pre- and post-bicycle adjustments, a Student’s *t*-test was employed. Cohen’s *d* effect sizes were determined using a custom script math program to determine the extent of the differences. It was stablished as small, medium, and large effect sizes for standardized mean differences of 0.2, 0.5, and 0.8 [41]. The statistical significance threshold was established at alpha level 0.05 for all data, using the Statistical Package for Social Sciences (SPSS) v. 20 (IBM, Chicago, IL, USA) and Jamovi Statistical Software v10.13 (Sydney, Australia) programs.

## 3. Results

**Figure 3:**
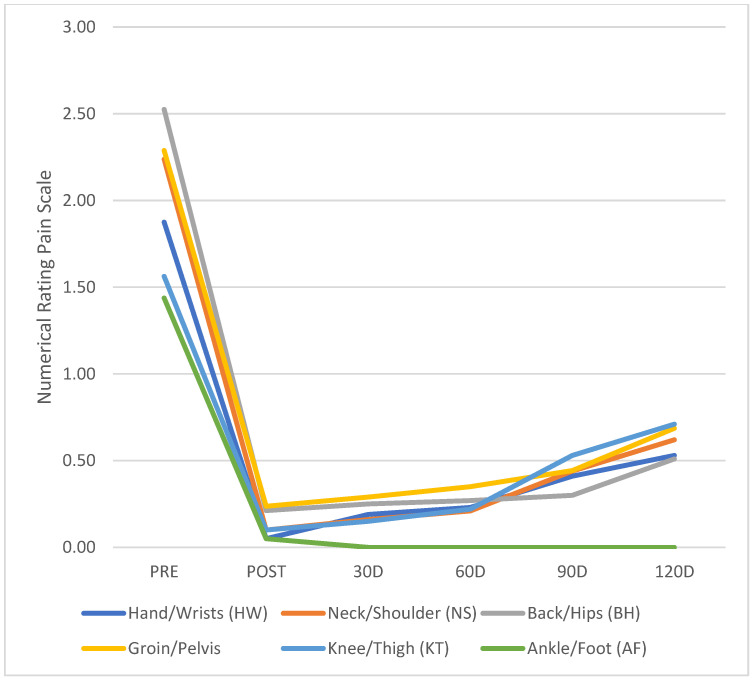
Numerical Rating Pain Scale Values of All Five Body Parts at Each Time Point.

**Figure 4:**
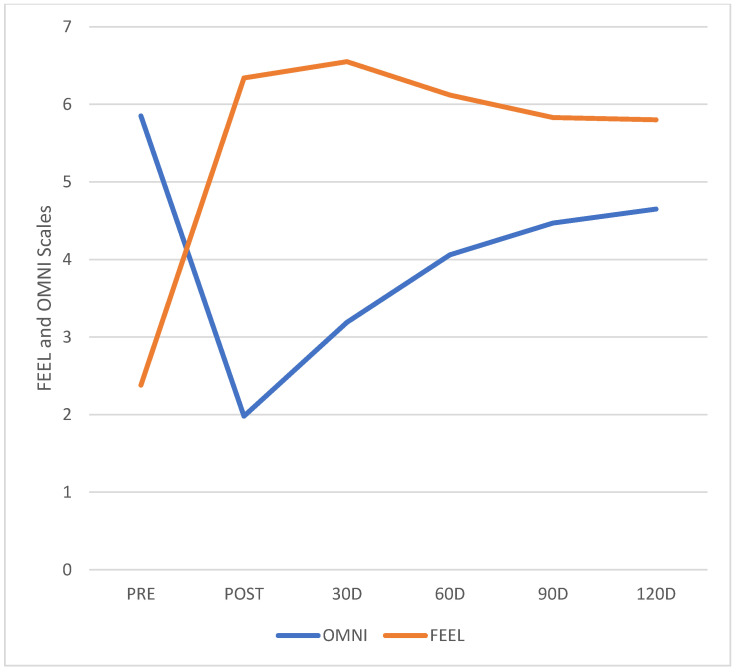
FEEL and OMNI Scales Values at Each Time Point.

The descriptive analysis results of all variables are presented in Table 3, while its respective graphical plots are illustrated in Figure 3 and Figure 4. It shows the means and standard deviations of all outcome measures under analysis (riding pain from NRPS Scale, riding fatigue from OMNI Scale, and riding discomfort from FEEL Scale) in all six time points.

Inferential analysis results are presented in Table 4. It shows the statistical differences between all variables under analysis at two time points: pre-intervention and post 120 days of intervention along with its confidence intervals and effect sizes.

Figure 3 illustrates the behavior of all pain-related variables along 4 months of follow-up. It shows a significant reduction of all NRPS variables just after intervention, followed by a small and non-significant increase after 120 days.

Figure 4 illustrates the behavior of both riding fatigue and riding discomfort values along 4 months of follow-up. It shows a significant change in both variables at post-intervention time points. However, after 120 days, OMNI values increased, and their difference from pre-intervention values became non-significant.

The results show that all eight variables showed statistically significant differences between time points PRE and 120 days after the bikefit session (*p* < 0.05). In addition, there was a very large effect size for the Feeling Scale and OMNI Scale changes (1.18 and 1.99, respectively). Pain reduction in the knee/thigh, back/hips, and shoulders/neck regions also had a large effect size (0.78, 0.72, and 0.73, respectively).

## 4. Discussion

In summary, our findings showed that a standardized 3D kinematic-oriented bikefitting process can reduce riding pain, fatigue, and discomfort for as long as 4 months post intervention, as all eight variables under analysis showed statistically significant differences between time points PRE and 120 days after the bikefit session. In addition, there was a very large effect size for the Feeling Scale and OMNI Scale changes, indicating a significant clinical relevance. Riding pain reduction in the knee/thigh, back/hips, and shoulders/neck regions showed large effect sizes, also indicating a significant clinical relevance.

The aim of this study was to analyze the cyclist’s long-term subjective responses to an ergonomic bicycle fit protocol performed on their own bikes through a kinematically guided bikefit session. Although we could not find, in the scientific literature, evidence or consensus about the adequate timeframe for a long-term concept of bikefitting effects duration, in the absence of a hard evidence on this matter, we defined a period of 4 months (120 day) as an adequate duration based on the data of interviews with participants of our other studies regarding bikefitting [28,35]. We assumed that mountain bikers riding more than 200 km per month usually change some of their bicycle components three times a year.

Our hypothesis was that subjective levels of pain, discomfort, and fatigue would continue to be significantly lower even after 120 days of intervention. This hypothesis was partially confirmed by our findings, as all eight variables showed statistically significant differences between the first data collection (pre-intervention) and the last data collection 4 months after (*p* < 0.05). We took care to emphasize to all participants that they should keep the final bicycle configuration stablished after the bikefit session, avoiding additional adjustments at home or at the local mechanical bike shop. However, some changes could have occurred (even without owner´s knowledge) on their bicycle’s configuration during this large time frame, as mountain bikes are frequently disassembled for transport, maintenance, and repairs. Every bikefit kinematic process made in our study generated a report with all bicycle configurations. These reports were given and explained to participants in the attempt to reduce this bias although after 120 days, we did not have the opportunity to confirm each bicycle configuration in person.

The fatigue values reported by our participants showed a tendency to return to baseline values after three months of intervention. The other variables also showed a tendency to increase their values over the passage of months; however, at the end of 120 days, they still remained significantly lower than baseline values. As mentioned before, to the best of our knowledge, this is the first study to evaluate the effects of bikefitting over a long period of time, and thus, we cannot make comparisons with other studies. However, we can make assumptions based on parallel data extracted from the same sample. Hence, we performed a post hoc Pearson’s correlation analysis between riding pain, comfort, fatigue, and monthly training (riding) volume. From this analysis, we found a high correlation between fatigue degree and the current training volume in kilometers over time (Pearson’s correlation, *r* = 0.946). This leads us to believe that, with the increase in riding comfort (showed by reduced Feeling Scale values below baseline at all time points), cyclists gradually increasing their weekly mileage—consequently increasing their fatigue scores while practicing their sport.

Although still significantly lower after 120 days, pain scores around the knee and groin also showed a small increase. As occurred with fatigue, we performed a post hoc Pearson’s correlation between pain scores and monthly training volume, and it was not surprising to us that both variables also showed an association (Pearson’s correlation, r = 0.532) with increased training volume, resulting in a moderate relationship. Thus, this increased pain could also be (at least partially) explained by the increased mileage. According to several authors, the knees and groin areas are often targets of non-traumatic musculoskeletal pain by cyclists imperfectly fitted to their bicycles or by high-volume riders such as professional athletes [13,20,21,42,43,44].

The degree of improvement in pedaling comfort (measured with the Feeling Scale) resulted in a large effect size, indicating an important clinical relevance, mostly to sports professionals interested in bikefitting, as riding discomfort is an important contributor to the adherence of recreational cyclists to their sports practice [4,8,28].

Localized pain intensity had a moderate effect size in its reduced values in the five body parts of all participants, while a small effect size in pain reduction was detected around ankle and foot body regions. These results are in agreement with other cycling pain studies by other authors [6,8,28,45,46,47,48,49,50,51,52]. Few studies [1,6,8,28] have detailed information of riding pain of specific body parts (like our study) but instead generalized pain simply as “riding pain”. From those allowing comparison, our results confirm a moderate effect size on pain reduction post bikefitting, with knee, groin and back pain being the most frequent riding-related complaints.

As mentioned before, the absence of a control group limits our conclusions, as placebo effects could not be measured through comparison. Although a complete blind process seems impossible to perform (as the real bikefit procedure is evident to participants), a control group using minimal intervention could clarify at least in part the impact of placebo influence over the results. A second limitation of our study is to consider a period of 120 days as long term. Even after several bibliographic searches on scientific databases, we could not find agreement between researchers on the exact timeframe of what could be considered short term and long term for ergonomic adjustments on cycling. This should be assessed in future studies.

## 5. Conclusions

Overall, riding discomfort and pain were significantly decreased after a standardized kinematic bikefit session even after 120 days post intervention. However, fatigue scores began to rise after 30 days, showing a high correlation with increasing monthly training volume.

In practice, professional bikefitters, cyclists, and coaches may use these findings to improve their overall pedaling experience with scientifically based bikefitting data (using any kinematic measurement tool) in contrast to anecdotal orientations. In our study, fitting 15 angle ranges is enough to produce large and long-term effects on riding pain, fatigue, and comfort.

To increase overall riding comfort and lessen pain, mountain bike ergonomic changes may be made using the suggested angular ranges that were employed in our study. Future research should use the three subjective scales that were used in this study in order to draw accurate conclusions about the clinical applicability of their findings.

## Figures and Tables

**Table 1:** Demographic and anthropometric characteristics of all participants (Means ± Standard Deviation).

Participants (*n* = %)	
Male (*n* = %)	54 = 67.5%
Female (*n* = %)	26 = 32.5%
Total (*n* = %)	80 = 100%
Age (years)	39.03 ± 5.11
Height (cm)	173.83 ± 7.82
Wingspan (cm)	174.68 ± 8.01
Body mass (kg)	76.96 ± 11.68
BMI (kg/m^2^)	25.35 ± 2.65
Familiarity with Current Bicycle (*n* = %)	
6 to 12 Months	36 = 45.0%
13 to 18 Months	11 = 13.8%
19 to 24 Months	14 = 17.5%
25+ Months	19 = 23.8%
Training Volume (*n* = %)	
60–120 km/month	3 = 3.8%
121–240 km/month	16 = 20.0%
241–480 km/month	21 = 26.3%
481–800 km/month	19 = 23.8%
800+ km/month	21 = 23.6%

Notes: BMI (body mass index).

**Table 2:** Recommended measurements and joint angular ranges for cycling 3D kinematics analysis. Quoted from our previous bikefitting study [28].

Measurement	Abbreviation	Angular Range	Description
Ankle minimum	AR.Min	65 to 75	Maximum dorsiflexion at any point in the pedal stroke defined by the knee-ankle line and the heel-foot line.
Ankle maximum	AR.Max	90 to 100	Maximum plantarflexion at any point in the pedal stroke defined by the knee-ankle line and the heel-foot line.
Ankle range	AR	20 to 30	The difference between ankle maximum and ankle minimum.
Ankle angle at bottom	AAB	90 to 100	The ankle angle at the bottom of the pedal stroke (180 degrees).
Maximum knee flexion	MKF	107 to 113	Maximum flexion of the knee joint at any point in the pedal stroke defined by the hip-knee line and the knee-ankle line.
Maximum knee extension	MKE	32 to 42	Maximum extension of the knee joint at any point in the pedal stroke defined by the hip-knee line and the knee-ankle line.
Knee angle range	KAR	70 to 75	The difference between knee angle flexion and knee angle extension.
Knee forward of foot	KFF	−10 to 10	The fore/aft offset of the knee marker relative to the foot marker captured at the forward part of the pedal stroke (3 o’clock or 90 degrees down). A negative number indicates a knee that is aft of neutral.
Knee forward of spindle	KFS	−35 to −5	The fore/aft offset of the knee marker relative to the pedal spindle at 3 o’clock in the pedal stroke (90 degrees in the downstroke).
Knee travel tilt	KTT	−2 to 4	The frontal plane angle of the tracing created by the moving knee marker with respect to vertical. A positive number indicates a knee that tracks away from the bike in the upstroke. A negative number represents a knee that tracks towards the bike in the upstroke. See the front view of the knee path for a visual representation of this measurement.
Knee lateral travel	KLT	5 to 36	The magnitude of the lateral movement of the knee.
Hip angle closed	HAC	66 to 76	The most closed angle of the hip joint defined by the knee, hip, and shoulder marker.
Hip angle open	HAO	110 to 120	The most open angle of the hip joint defined by the knee, hip, and shoulder marker.
Hip angle range	HAR	40 to 45	The difference between hip angle open and closed.
Hip lateral travel	HLT	5 to 20	The magnitude of the lateral movement of the hip
Back angle	BA	50 to 65	The angle of the back relative to the horizon defined by the hip and shoulder marker
Shoulder angle to wrist	SAW	65 to 75	The angle of the shoulder joint defined by the hip, shoulder, and wrist markers.
Shoulder angle to elbow	SAE	60 to 70	The angle of the shoulder joint defined by the hip, shoulder, and elbow markers.

**Table 3:** Descriptive analysis of all variables.

Variable	Mean	SD	SE
NRPS.HW.Pre	1.88	1.26	0.14
NRPS.HW.Post	0.05	0.22	0.02
NRPS.HW.30 Days	0.19	0.20	0.8
NRPS.HW.60 Days	0.23	0.18	0.05
NRPS.HW.90 Days	0.41	0.31	0.11
NRPS.HW.120 Days	0.53	0.40	0.21
NRPS.NS.Pre	2.24	1.69	0.19
NRPS.NS.Post	0.10	0.44	0.05
NRPS.NS.30 Days	0.16	0.35	0.06
NRPS.NS.60 Days	0.21	0.51	0.09
NRPS.NS.90 Days	0.44	0.67	0.11
NRPS.NS. 120 Days	0.62	0.79	0.23
NRPS.BH.Pre	2.53	2.06	0.23
NRPS.BH.Post	0.21	0.63	0.07
NRPS.BH.30 Days	0.25	0.37	0.11
NRPS.BH.60 Days	0.27	0.41	0.16
NRPS.BH.90 Days	0.30	0.43	0.13
NRPS.BH.120 Days	0.51	0.62	0.15
NRPS.GP.Pre	2.29	1.73	0.19
NRPS.GP.Post	0.24	0.75	0.08
NRPS.GP.30 Days	0.29	0.88	0.11
NRPS.GP.60 Days	0.35	0.98	0.14
NRPS.GP.90 Days	0.44	1.05	0.12
NRPS.GP.120 Days	0.69	1.12	0.22
NRPS.KT.Pre	1.56	1.96	0.22
NRPS.KT.Post	0.10	0.44	0.05
NRPS.KT.30 Days	0.15	0.32	0.10
NRPS.KT.60 Days	0.22	0.40	0.14
NRPS.KT.90 Days	0.53	0.68	0.15
NRPS.KT.120 Days	0.71	0.99	0.22
NRPS.AF.Pre	1.44	1.04	0.12
NRPS.AF.Post	0.05	0.35	0.04
NRPS.AF.30 Days	0.00	0.00	0.00
NRPS.AF.60 Days	0.00	0.00	0.00
NRPS.AF.90 Days	0.00	0.00	0.00
NRPS.AF.120 Days	0.00	0.00	0.00
FEEL.Pre	2.38	1.63	0.18
FEEL.Post	6.34	1.74	0.19
FEEL.30 Days	6.55	2.46	0.23
FEEL.60 Days	6.12	2.38	0.20
FEEL.90 Days	5.83	2.35	0.25
FEEL.120 Days	5.80	2.29	0.21
OMNI.Pre	5.85	1.21	0.14
OMNI.Post	1.98	1.49	0.17
OMNI.30 Days	3.19	1.16	0.33
OMNI.60 Days	4.06	1.21	0.27
OMNI.90 Days	4.47	1.48	0.30
OMNI.120 Days	4.65	1.74	0.41

Notes: Standard deviation (SD), standard error (SE), Numerical Rating Pain Scale (NRPS), Feeling Scale (FEEL), OMNI scale (OMNI), hand/wrists (HW), neck/shoulders (NS), back/hips (BH), groin/pelvis (GP), knee/thigh (KT), ankle/foot (AF).

**Table 4:** Inferential analysis showing the mean differences between pre and post intervention of each group with its respective effect size.

Paired Samples *t*-Test		Statistic	*p*	Mean Difference	SE Difference	95% CI		Cohen’s d Effect Size	95% CI	
						Lower	Upper		Lower	Upper
NRPS.HW.Pre	NRPS.HW.120D	6.10	<0.001 *	0.83	0.14	0.56	1.09	0.68	0.44	0.92
NRPS.NS.Pre	NRPS.NS.120D	6.53	<0.001 *	1.14	0.17	0.79	1.48	0.73	0.48	0.98
NRPS.BH.Pre	NRPS.BH.IG.Post	6.48	<0.001 *	1.31	0.20	0.91	1.72	0.72	0.48	0.97
NRPS.GP.Pre	NRPS.GP.IG.Post	5.12	<0.001 *	0.95	0.19	0.58	1.32	0.57	0.33	0.81
NRPS.KT.Pre	NRPS.KT.IG.Post	6.98	<0.001 *	1.46	0.21	1.05	1.88	0.78	0.53	1.03
NRPS.AF.Pre	NRPS.AF.IG.Post	3.16	0.002 *	0.39	0.12	0.14	0.63	0.35	0.13	0.58
FEEL.Pre	FEEL.IG.Post	−10.52	<0.001 *	−2.96	0.28	−3.52	−2.40	−1.18	−1.46	−0.89
OMNI.Pre	OMNI.IG.Post	17.83	<0.001 *	3.48	0.20	3.09	3.86	1.99	1.61	2.37

Notes: Standard deviation (SD), standard error (SE), Numerical Rating Pain Scale (NRPS), Feeling Scale (FEEL), OMNI scale (OMNI), hand/wrists (HW), neck/shoulders (NS), back/hips (BH), groin/pelvis (GP), knee/thigh (KT), ankle/foot (AF), statistically significant (*), confidence interval (CI).
